# Favorable one-year outcomes despite residual fascial tension after ventral hernia repair with transversus abdominis release

**DOI:** 10.1007/s10029-026-03755-y

**Published:** 2026-06-15

**Authors:** Jay J. W. Han, William C. Bennett, Jalen M. Plaster, Ryan C. Ellis, Alvaro C. Carvalho, Jennifer Lee, Luciano Tastaldi, Lucas R. Beffa, David M. Krpata, Ajita S. Prabhu, Clayton C. Petro, Michael J. Rosen, Benjamin T. Miller

**Affiliations:** 1 Case Western Reserve University School of Medicine, Cleveland, OH USA; 2 Center for Abdominal Core Health, Department of General Surgery, Digestive Disease Institute, Cleveland Clinic Foundation, Cleveland, OH USA; 3 Cleveland Clinic Lerner College of Medicine at the Case Western Reserve University School of Medicine, Cleveland, OH USA

**Keywords:** Ventral hernia, Transversus abdominis release, TAR, Tension, Component separation, Herniorrhaphy

## Abstract

**Purpose:**

Fascial tension is thought to adversely impact herniorrhaphy, but the impact of residual tension on outcomes is uncharacterized beyond the immediate postoperative period. We previously reported fascial tension changes in a case series of patients who underwent posterior component separation with transversus abdominis release (PCS-TAR) and found no association between anterior fascia closure tension and early surgical outcomes. Here we consider patient outcomes at and beyond one postoperative year.

**Methods:**

In this post-hoc analysis of a previously reported case series, surgical and patient-reported outcomes beyond one year were compared to the force (lbs) required to medialize anterior fascial elements before and after PCS-TAR. Patient demographics and surgical outcomes were captured in the Abdominal Core Health Quality Collaborative (ACHQC) registry, which was queried to identify surgical outcomes, including recurrence, wound morbidity, and patient-reported quality of life (QoL; hernia-specific and pain). Univariate regressions tested strength of association between fascial tension measures (baseline, closure, and intraoperative delta) and outcomes of interest.

**Results:**

Of the 100 patients in the original case series, long-term clinical or patient-reported follow-up was available for 77. The long-term cohort had a median hernia width of 13 cm (IQR 10, 15), median baseline fascial tension of 11 lbs (IQR 6, 19) and median closure tension of 4 lbs (IQR 2, 8). Recurrence rate was 1.4% (*n* = 1) among those with clinical follow-up. No tension measures were associated with recurrence, wound morbidity, or patient-reported outcomes.

**Conclusions:**

In this exploratory analysis of 77 abdominal wall reconstruction operations with PCS-TAR, a near absence of adverse outcomes were observed at one year among cases with a median residual midline closure tension of 4 lbs. Hernia-specific QoL and pain were not associated increased closure tension. The analyses are markedly limited by low event rates but tentatively suggest safety within the observed range of residual tension measurements. Larger cohorts featuring higher tension measurements are necessary to substantiate the findings and identify an upper limit of ‘safe’ residual tension.

**Supplementary Information:**

The online version contains supplementary material available at 10.1007/s10029-026-03755-y.

## Introduction

Fascial tension has long been considered deleterious in the context of ventral hernia repair, although objective measurements of tension and associated outcomes are scarcely described in hernia literature [1]. Increased closure tension is thought to be associated with both early and late adverse outcomes such as impaired wound healing, respiratory complications, abdominal compartment syndrome, and increased risk of hernia recurrence [2–5]. Several techniques have been employed to facilitate fascial closure by decreasing midline tension and increasing abdominal wall compliance, such as component separation or use of preoperative adjunctive techniques including neuromuscular toxin injection or progressive pneumatic peritoneal expansion [6]. However, few objective data guide surgical decision-making regarding residual tension and/or appropriate utilization of techniques aimed at decreasing tension at time of closure.

Previously, our group quantified tension at the time of fascial closure after ventral hernia repair with posterior component separation and transversus abdominis release (PCS-TAR) and described an absence of adverse outcomes associated with residual closure tension at 30 days [7, 8]. In this study we aimed to describe long-term clinical outcomes associated with fascial tension during fascial reapproximation or closure at the time of PCS-TAR.

## Methods

This study was a post-hoc analysis of a prospective case series that measured fascial tension in the abdominal wall before and after PCS-TAR. Institutional Review Board approval was obtained prior to any data collection or analysis. The measurement of fascial tension and corresponding findings have been previously reported [7, 8]. To summarize, the original study enrolled 100 adult patients with European Hernia Society (EHS) classification M1 to M5 ventral hernias who underwent PCS-TAR at the Cleveland Clinic Center for Abdominal Core Health (NCT05142761) from January 2022 to July 2022. Patients who had stomas, previously undergone PCS, or were categorized as EHS L1 to L4 were excluded from this study. Informed consent was required and collected for all participants prior to participation in the original study. This post-hoc analysis was approved without requiring additional consent discussion or collection, as all follow-up and patient contact were conducted in a manner consistent with our standard institutional practices and all study data were utilized in a manner consistent with the existing consent document and prior informed consent discussion(s).

**Fig. 1:**
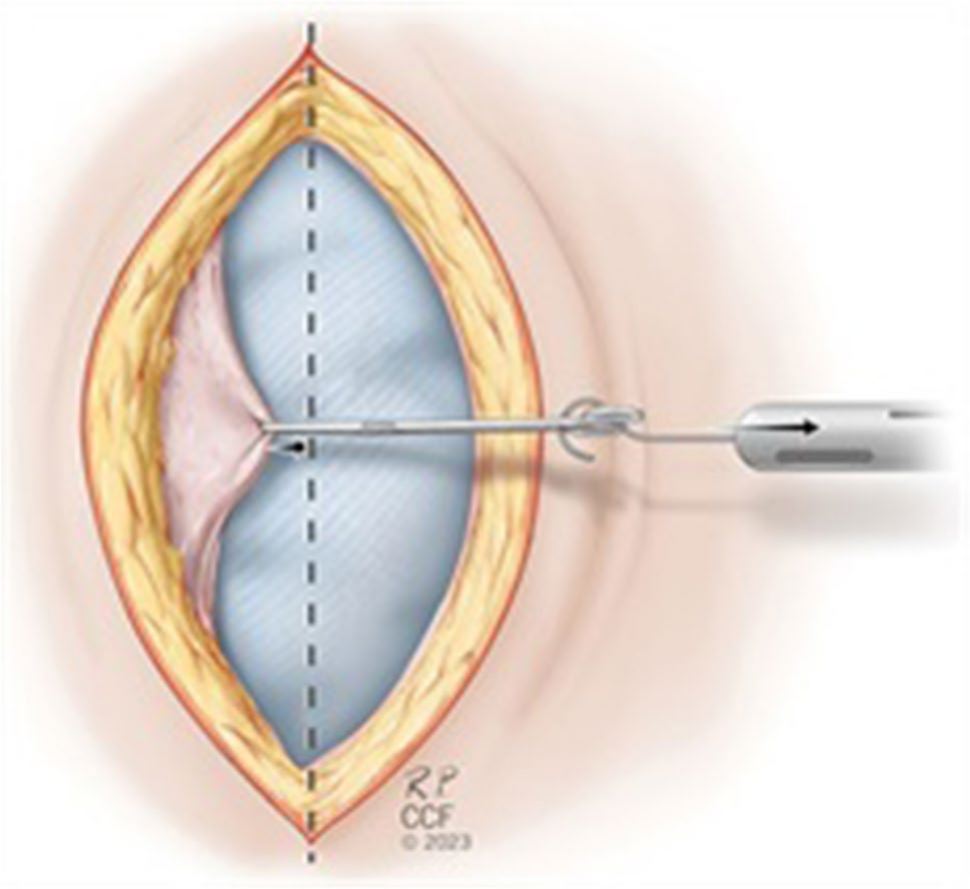
Abdominal wall tensiometry. Originally published by Miller et al. [7]. Reprinted with permission, Cleveland Clinic Foundation ©2024. All Rights Reserved

All participants enrolled in the index study underwent PCS-TAR as described by Krpata et al. [9]. Following complete adhesiolysis, bilateral baseline fascial tension was measured. Anterior fascial tension was recorded at multiple time points for each patient; in this study we consider baseline tension (prior to retrorectus release) and tension at closure (following TAR). Tension was measured as previously described [7, 8]. Briefly, a tensiometer was connected to a clamp on the fascial edge at the level of greatest defect width. The tensiometer was then pulled until fascia was medialized and the tension in pounds (lbs) was recorded with a range of 0–15 lbs (Fig. 1). Tension from each side was summed for a single bilateral tension value. After PCS-TAR and placement of mesh in the retromuscular space, the anterior fascia was closed using #1 polydiaxanone suture in either a running or interrupted figure-of-eight fashion.

Standard of care follow-up was conducted for all participants as per our routine practice, in which patient-reported outcomes and clinical outcomes are captured and recorded in the Abdominal Core Health Quality Collaborative (ACHQC) registry at annual intervals [10]. The ACHQC is a hernia-based registry that collects patient-reported outcomes and long-term follow-up to improve the quality of abdominal core and hernia care. Standard annual follow-up is preferentially conducted in-person. Participants without in-person follow-up are contacted via email or phone; no more than three attempts are made per patient, per interval. During such follow-up the hernia-related quality-of-life survey (HerQLes), Patient-reported Outcomes Measurement Information System Pain Intensity Short Form 3a (PROMIS 3a), and ventral hernia recurrence inventory (VHRI) surveys are administered. Simultaneously, the electronic medical record (EMR) is reviewed for supplementary data including cross-sectional imaging, clinical exams, operative reports, and other clinical notes to identify instances of wound morbidity, recurrence, and reoperation.

Patients found to have had subsequent abdominal surgery for an unrelated pathology were excluded from analysis, as this violation of the repair would confound the association between closure tension and long-term outcomes. Outcomes prior to any such operation were included if captured at an appropriate time interval.

Tension was evaluated with respect to outcomes, including wound morbidity and recurrence, at and after 1-year. Wound morbidity, including surgical site infection (SSI), surgical site occurrence (SSO), and surgical site occurrence requiring procedural intervention (SSOPI), was diagnosed using standardized definitions for postoperative wound events after ventral hernia repair [11]. Hernia recurrence was derived from the most recent ACHQC recurrence evaluation for each participant and defined in a hierarchical fashion. Radiographic recurrence evaluations within the ACHQC were considered the highest fidelity indicator of recurrence (reoperation for recurrence would otherwise typically be considered first but was not observed in this cohort). All radiographs were reviewed by the operating surgeon utilizing a recurrence definition of “a protrusion of abdominal contents across a fascial defect within 7 cm of the original repair.” Surgeons were blinded to hernia size and tension measures at time of image. Recurrence evaluation via clinical exam was utilized as the second tier of recurrence evidence. If clinical exam and radiographic data were both available and discordant at the same timepoint, the radiographic outcome was utilized. An additional definition of recurrence— pragmatic recurrence— was also considered. The definition incorporates patient-reported response to the “do you feel or see a bulge” item within the VHRI as a third tier of recurrence information. The pragmatic definition previously described by Krpata et al. was utilized in addition to the primary definition and is reproduced in the supplemental document for ease of reference (Supplemental eTable 1) [12]. The pragmatic definition of recurrence was kept separate from the primary definition given recent concerns over validity of the ‘bulge’ item for reliably detecting ventral hernia recurrence. [13] Hernia-specific quality of life was measured via HerQLes survey administered no sooner than one year postoperatively. The HerQLes is a twelve-item questionnaire validated in our patient population; the scaled score ranges from 0 to 100 with a higher score indicating a higher quality of life [14]. Pain was measured via PROMIS 3a, which is a validated survey to capture all-cause patient-experienced pain; responses are converted to t-scores ranging from 30.7 to 71.8 where higher scores indicate more pain [15, 16].

### Statistical analysis

Continuous variables were summarized using median and interquartile range (IQR), and categorical variables were summarized using counts and percentages. HerQLes was analyzed both as a continuous measure and dichotomized at a prespecified threshold of 40 (≥ 40 vs < 40), consistent with prior literature deeming this as a cut-off for low quality of life [17]. PROMIS T-scores were similarly analyzed as continuous variables and dichotomized at a threshold of 35.07 to distinguish those without any detectable pain vs those reporting some degree of pain.

To evaluate the association between abdominal wall tension and postoperative outcomes, univariate logistic regression models were used to examine the effect of baseline tension, tension at closure, and change in tension vs binary outcomes, including recurrence, pragmatic recurrence, patient-reported bulge, dichotomized HerQLes, and dichotomized PROMIS T-score. In addition, simple linear regression models were used to assess the relationship between tension measures and continuous HerQLes and PROMIS T-scores.

Given the low number of recurrence events, all regression analyses were exploratory and unadjusted. In lieu of adjusted analyses, color-coded jittered boxplots were generated to provide context and demonstrate distribution patterns of hernia and mesh dimensions (width and length) as they related to tension measures and clinical outcomes of interest (recurrence, pragmatic recurrence, VHRI bulge item response, HerQLes summary score, PROMIS 3a T-score). Preliminary association testing between each measurement and the tension or outcome measure plotted were conducted via Spearman rank test for continuous variables and Wilcoxon rank-sum test for binary outcomes, with corresponding p-values on each panel. Statistical analyses were performed using RStudio. A two-sided p-value < 0.05 was considered statistically significant.

## Results

The updated analytic cohort included 77 of 94 eligible adult patients (81.9%). Of the 100 participants in the original study, two died prior to one year and four underwent early reoperation unrelated to hernia repair. Demographics for the long-term cohort were similar to the original cohort. Median age was 61 years (IQR 53.5, 68.5), 33 (42.9%) were male, median BMI was 33 kg/m^2^ (IQR 29.7, 35.9) (Table 1). Primary fascial closure was achieved in all patients (*n* = 77, 100%). Median hernia width and length were 13 (IQR 10, 15) cm and 23 (IQR 19, 25) cm, respectively (Table 2). Median baseline anterior fascial tension was 11 (IQR 6, 19) lbs, median closure tension was 4 (IQR 2, 8) lbs, and median change was −5.5 (IQR −9, −2.5) lbs.

**Table 1:** Patient demographics, comorbid conditions, hernia, and wound characteristics

Characteristic	Total (*N* = 77)
Female Sex, n (%)	33 (43%)
Age, y, median [IQR]	61 (54, 68)
BMI, kg/m^2^, median [IQR]	33.0 (29.7, 35.9)
Obesity (BMI > 30 kg/m^2^), n (%)	56 (73%)
Hypertension, n (%)	43 (56%)
Current smoker, n (%)	4 (5.2%)
ASA Classification, n (%)	
II	15 (19%)
III	61 (79%)
IV	1 (1.3%)
Diabetes, n (%)	19 (25%)
COPD, n (%)	7 (9.1%)
Congestive heart failure, n (%)	3 (3.9%)
Inflammatory bowel disease, n (%)	4 (5.2%)
Immunosuppression, n (%)	5 (6.5%)
Recurrent incisional hernia, n (%)	41 (53%)
History of abdominal wall infection, n (%)	17 (22%)

IQR, interquartile range (IQR1, IQR3). BMI, body mass index. ASA, American Society of Anesthesiologists. COPD, chronic obstructive pulmonary disease

**Table 2:** Summary of operative details

Operative Characteristic	Total (*N* = 77)
CDC wound class, n (%)	
I	63 (82%)
II	13 (17%)
III	1 (1.3%)
Operating room time, n (%)	
0–59 min	0 (0%)
60–119 min	18 (23%)
120–179 min	25 (32%)
180–239 min	20 (26%)
240 + min	14 (18%)
Hernia width, cm, median [IQR]	13.0 (10.0, 15.0)
Hernia length, cm, median [IQR]	23.0 (19.0, 25.0)
Mesh width, cm, median [IQR]	30 (30, 42)
Mesh length, cm, median [IQR]	30 (30, 40)
Anterior fascial closure, n (%)	77 (100%)
Enterotomy, n (%)	1 (1.3%)
Concomitant procedure, n (%)	10 (13%)
Gastrointestinal	7 (9.1%)
Gynecologic	1 (1.3%)
Plastics	2 (2.6%)
Baseline Tension	11 (6, 19)
Tension at closure	4.0 (2.0, 8.0)
Change in tension	−6.3 (−10.0, −3.5)

*CDC* center for disease control; *IQR* interquartile range (IQR1-IQR3)

### Follow-up and clinical outcomes

Clinical follow-up at ≥ 1-year was obtained for 67 of included patients (87%) and patient-reported outcomes were available for 51 (66%). Median time to follow-up was 1,135 days (Table 3).

**Table 3:** Description of available follow-up for participants at or beyond one year postoperatively and summary statistics of captured outcome

Characteristic	Total (*N* = 77)
Clinical Exam, n (%)	67 (87%)
Radiographic Exam, n (%)	45 (58%)
Clinical OR Radiographic Exam, n (%)	69 (90%)
PRO, n (%)	49 (64%)
Days between operation and last follow-up, median [IQR]	1,135 (922, 1,208)
Recurrence, n (%)	1 (1.4%)
Pragmatic Recurrence, n (%)	2 (2.6%)
PRO Bulge, n (%)	10 (20%)
HerQLeS scaled, median [IQR]	88 (50, 92)
PROMIS 3a (v1.0) t-score, median [IQR]	31 (31, 40)

*PRO* patient-reported outcome; *IQR* interquartile range; *HerQLes* hernia-related quality of life survey; *PROMIS* patient-reported outcome measurement information system

No SSI, SSO, or SSOPIs were identified at or beyond one year for any participant. Nor was reoperation for recurrence. Hernia recurrence was rare, with one patient (1.5%) found to have a clinical recurrence among 67 patients with cross-sectional imaging or physician physical exam. This patient had a baseline tension of 15.5 lbs and closure tension of 7 lbs, associated with a 12 cm wide and 23 cm long hernia. Univariate logistic regression found no association between odds of clinical recurrence and any tension measure (Table 4).

**Table 4:** Univariate logistic regression model of the effect of abdominal wall tension on selected follow-up outcomes

Outcomes	Baseline Tension		Tension at Closure		Change in Tension		
	OR	95% CI	OR	95% CI	OR		95% CI
Recurrence	1.03	[0.78, 1.31]	1.02	[0.62, 1.26]	0.95	[0.65, 1.54]	
Pragmatic Recurrence	0.94	[0.70, 1.12]	0.91	[0.53, 1.15]	1.07	[0.81, 1.66]	
PRO Bulge	0.90	[0.78, 1.01]	0.87	[0.66, 1.03]	1.13	[0.95, 1.40]	
HerQLes Scaled	1.04	[0.92, 1.17]	1.07	[0.91, 1.23]	1.01	[0.81, 1.34]	
PROMIS 3a T-score	1.01	[0.94,1.09]	0.96	[0.84, 1.06]	0.90	[0.77, 1.03]	

*OR* odds ratio

### Patient-reported outcomes

**Fig. 2:**
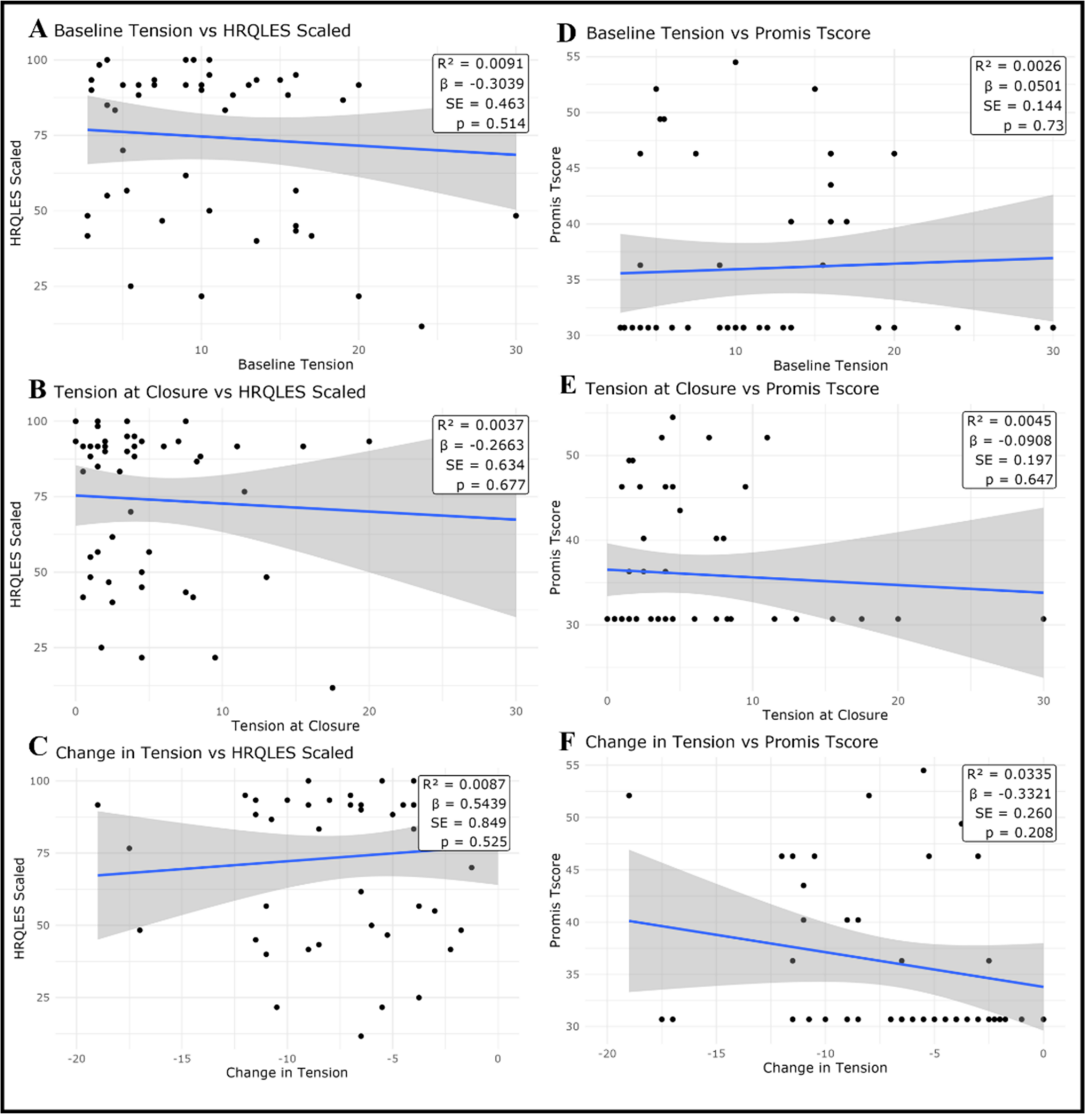
Univariate linear regressions of HerQLes summary score and PROMIS 3a T-score vs baseline fascial tension (A and D), closure tension (B and E), and change in tension (C and F), respectively

HerQLes and PROMIS 3a responses were available for 49 participants, and a patient-reported bulge response was available for an additional two (*n* = 51). Patient-reported quality of life was generally favorable, with a high median HerQLes score (88.33 (IQR) 49.2, 92.5), indicating good health-related quality of life and low symptom burden. Only four patients met criteria for ‘low’ quality of life, defined as ≤ 40. No association between HerQLes scores and tension measures (baseline, closure, or change) was identified via linear regression (Fig. 2A-C), nor were associations between tension measures and low QoL (Table 4).

PROMIS 3a scores indicated low pain incidence with a median t-score of 30.7 (IQR 30.7, 41.85), which is the lowest possible score using the original scoring convention. Eighteen patients met the threshold for ‘any pain’ based on a definition of ≥ 36, which is the second lowest possible score (36%). Logistic regression similarly failed to identify any relationships between tension measurements and incidence of ‘any pain’ reported at long-term intervals (Table 4). Linear regression of t-scores found no association between long-term PROMIS scores and any tension measure (Fig. 2D-F).

Patient-reported bulge sensation, often used as a corollary for recurrence in lieu of clinical data, was reported by 10 patients among 51 responses (19.6%) and poorly corresponded with clinical recurrence detection. Nine of the 10 had clinical recurrence evaluations: Seven of them were found to have no recurrence on concurrent cross-sectional imaging and two had negative physical exams. Logistic regression identified no relationship between patient-reported bulge sensation and any of the tension measures (Table 4).

### Hernia and mesh dimensions

Distribution of relevant hernia repair dimensions (hernia width, hernia length, mesh width, and mesh length) for tension measures and outcomes are provided in Supplemental Figs. 1–8. Recurrence, pragmatic recurrence, VHRI ‘bulge’ response, and PROMIS 3a t-score were not associated with any dimension measurement. HerQLes scaled score was inversely correlated with increasing hernia length (rho = −0.31, *P* = 0.029), but not hernia width, mesh width, or mesh length (Supplemental Fig. 4). Baseline tension and tension at closure, but not change in tension, were correlated with hernia width, hernia length, mesh width, and mesh length (Supplemental Figs. 6–8).

## Discussion

In this follow-up case series of 77 patients who underwent large ventral hernia repair via PCS-TAR with complete fascial closure at least one year prior, we observed a near absence of adverse outcomes frequently associated with fascial closure under tension in a cohort featuring a median hernia width of 13 cm (IQR 10, 15) and median closure tension of 4 lbs (IQR 2.0, 8.125)— more than double the 1.94 lbs of tension required for midline fascial closure following midline laparotomy in patients without hernias [18]. Though restricted by low event rates, our analyses failed to identify any associations between residual fascial tension and other salient outcomes such patient-reported hernia-specific QoL or pain beyond the first postoperative year. Notably, no recurrences occurred among the ten patients with ≥ 10 lbs of closing tension, but the low recurrence rate (1.4%) of the cohort prevented meaningful regression of tension and recurrence.

The results agree with previously reported short-term outcomes for this cohort suggesting residual tension at the time of fascial closure is not associated with adverse outcomes, but are seemingly at odds with the surgical tenet mandating “tension-free” approximation of tissue during surgery, particularly in hernia repair [8]. Benefits of tension reduction have been emphasized by distant and contemporary surgical pioneers, alike [19, 20]. In particular, the nomenclature of a “tension-free repair”, now a near universal tenet within hernia surgery, was coined by Irving Lichtenstein in 1986 in the context of inguinal hernia repair [21, 22]. Lichtenstein stated the prime factor behind herniorrhaphy failure was suturing structures together under tension (specifically structures not normally in apposition) and that modern techniques allow elimination of tension [22]. The term and accompanying sentiment quickly spread across inguinal and ventral hernia repair literature and the notions remain prevalent in component separation literature where facilitation of a tension-free repair (and/or increasing the chances of a tension-free repair) is frequently cited as the principal benefit of PCS-TAR [23–25]. Despite appearing to contradict surgical canon, the results should not be interpreted as deeming tension inconsequential. The risks of undue tension are well-recognized (i.e. tissue edge ischemia, suture pull-through, and/or direct failure of suture material) and remain important considerations for hernia repair at-large [26]. Rather, these findings suggest hernia repair with retromuscular sublay reinforcement uniquely facilitates favorable outcomes despite remaining tension. PCS-TAR reduces tension and increases axial compliance, but tension is not altogether eliminated— remaining tension appears to be the rule rather than the exception [7].

We suspect placement of large mesh prostheses in the retromuscular space compensates for remnant tension and is the primary beneficial mechanism of PCS-TAR (rather than outright elimination of tension). Broad mesh-tissue interface equates to a large surface area of tissue ingrowth to stabilize the abdominal wall and resist midline separation. While the timeline of tissue ingrowth is poorly characterized for the retromuscular plane (most studies characterize intraperitoneal mesh incorporation), current literature suggests the process is rapid, achieving clinically significant strength benefits within two to twelve weeks [27–31].

If some degree of residual tension is acceptable, the indications for preoperative adjuncts such as botulinum toxin injection or progressive preoperative pneumoperitoneum (PPP)— often utilized with the intention of reducing closure tension— may require revisitation [32–34]. Our results demonstrate durable repairs in cases with remnant tension and without preoperative adjuncts; however, an important caveat should not be overlooked: these patients were anticipated to achieve primary fascial closure (and each did) without the use of preoperative adjuncts. Failure to achieve anterior fascial closure is associated with markedly increased rates of recurrence and diminished patient-reported outcomes [35]. Therefore, an important direction of future inquiry is identification of hernia and/or patient factors indicative of likely fascial non-closure. Adjuncts may facilitate otherwise unlikely fascial closure in such circumstances, but no reliable method yet predicts this reliably in large ventral hernias [36].

No reliable method yet predicts closure tension either, and even if one did, our results failed to identify a referenceable threshold of residual tension to be deemed safe or unsafe. This does not imply (and should not be interpreted to suggest) such a threshold does not exist. The cohort featured a median closure tension of 4 lbs (IQR 2.0, 8.125), which as previously mentioned, is just slightly more than double the tension observed in regular midline laparotomy closure [18]. The inability to detect a threshold of acceptable residual tension via analysis of this cohort likely not only relates to a low range of closure tension but the observation of lower-than-expected adverse outcome rates within a limited cohort. Notably, we observed only one recurrence via the primary definition and two recurrences via the pragmatic definition. Rates for hernia recurrence following TAR vary substantially based on follow-up time and definition, but these rates are lower than anticipated. In a prior series of 1203 patients who underwent PCS-TAR at the same referral center with at least 1 year of follow-up, the 1-year recurrence rate was 6% when using a radiographic definition (nearly identical to the definition employed for this study) and 10% for ≥ 1 year (when considering patients with 1–6 years of follow-up data); further, using the pragmatic definition, these rates were 10% and 26%, respectively [37]. It is not unreasonable to assume that increasing closure tension(s) should positively correlate with increased recurrence probability after some theoretical threshold, with this in mind, perhaps the range of tension encountered in this study was too low for a cohort featuring fewer than 100 participants.

Identification of a threshold for safe closure tension is important, and our results thus suggest two routes for future inquiry: (1) studies featuring large sample sizes with hernias anticipated to have low recurrence/adverse event rates such as those seen here and (2) studies with smaller cohorts featuring much larger hernias anticipated to have higher recurrence/adverse event rates. However, both approaches are more easily said than done and the difficulty of conducting such studies highlights the importance of this investigation despite its limitations. Outside of the original case series for this cohort, only a handful of studies have reported investigations of intraoperative tension measures and only two of them reported outcomes for a combined total of 75 patients; unfortunately, neither reliably reported patient-level tension measurements [3, 38–42]. Therefore, the results of this investigation are important early steps toward identifying the role of tension in ventral hernia repair and should be used to inform, design, and direct future investigations.

In addition to the previously addressed limitations, there are several other key considerations that must be acknowledged when interpreting the findings. First, the tensiometer used to measure fascial tension had a maximum measurement of 15 lbs, prohibiting discrimination of tension values above this threshold— notably preventing accurate calculation of changes in tension for those with summed baseline measurements ≥ 30 lbs. Additionally, 31.6% of recurrence outcomes were not based on objective radiographic findings but instead relied on physical exam alone. While clinical exam is likely to identify large hernias, prior investigation suggests low sensitivity (77%) and negative predictive value (77%) vs CT scans for incisional hernias— these numbers are even lower for obese patients (73% and 69%, respectively) and this case series cohort featured a median BMI of 33 kg/m^2^ [43]. Whether the result of limited follow-up or an actual absence of adverse outcome events (namely recurrence), the low event rates prevented multivariate, adjusted analyses. Interpretation must also be confined to retromuscular repairs featuring PCS-TAR which confers well-recognized mechanical advantages vs inlay and onlay mesh configurations [44]. It should be noted that the absence of recurrence in this cohort does not necessarily indicate anterior fascia integrity in the setting of retromuscular ventral hernia repair; it is possible that anterior fascial separation(s) occurred but midline reinforcement and wide lateral mesh overlap prevented observation of recurrence on non-Valsalva cross-sectional imaging. Therefore, the observed tensions could result in recurrence outside of the context of ventral hernia repairs without PCS-TAR. A dedicated image review study with a protocol found to reliably identify anterior fascial disruption (in the absence of mesh failure) would be necessary to truly associate (or fail to associate) the observed closure tensions with anterior fascial dehiscence. Finally, the study was conducted at a high-volume abdominal wall reconstruction center by surgeons with fellowship training. Therefore, these findings may not be generalizable outside the clinical context of this hospital system and its patient population.

## Conclusion

This exploratory analysis of residual fascial tension after PCS-TAR observed only one hernia recurrence at one-year and failed to associate residual tension with adverse patient outcomes. Further investigations are necessary to identify acceptable thresholds of residual tension. Pending such investigations, these early results suggest a truly “tension-free” closure was neither common nor required for successful hernia repair in this cohort.

## Supplementary Information

Below is the link to the electronic supplementary material.

Supplementary file1 (DOCX 3186 KB)

## Data Availability

Deidentified data supporting the findings of this study can be made available for reasonable requests by contacting the corresponding author. Any sharing of data will require a data use agreement between the requester and the Cleveland Clinic Foundation. Data shared will be limited to parameters established in the original study’s consent documentation.
