# Supplementary material for: Favorable one-year outcomes despite residual fascial tension after ventral hernia repair with transversus abdominis release
Source: Hernia. 2026 Jun 15;30(1):255. doi: 10.1007/s10029-026-03755-y (PMC13269521; doi:10.1007/s10029-026-03755-y)
Supplement: Supplementary file 1 — Supplementary file1 (DOCX 3186 KB) [file 10029_2026_3755_MOESM1_ESM.docx]

Supplemental Document


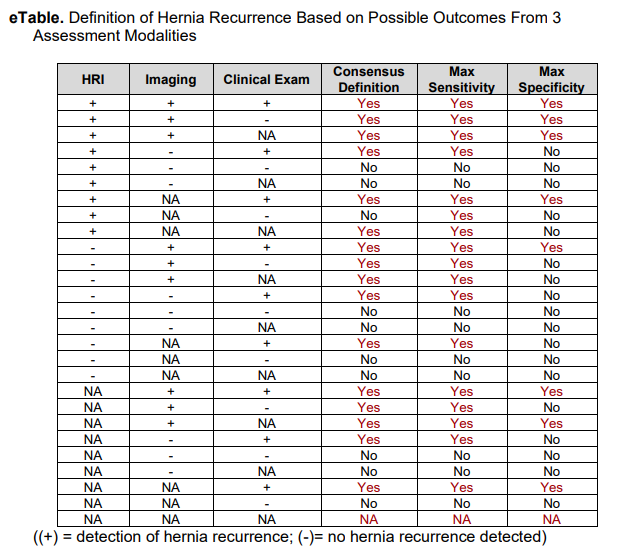


**Supplemental eTable 1. Algorithm to Determine Pragmatic Hernia Recurrence**


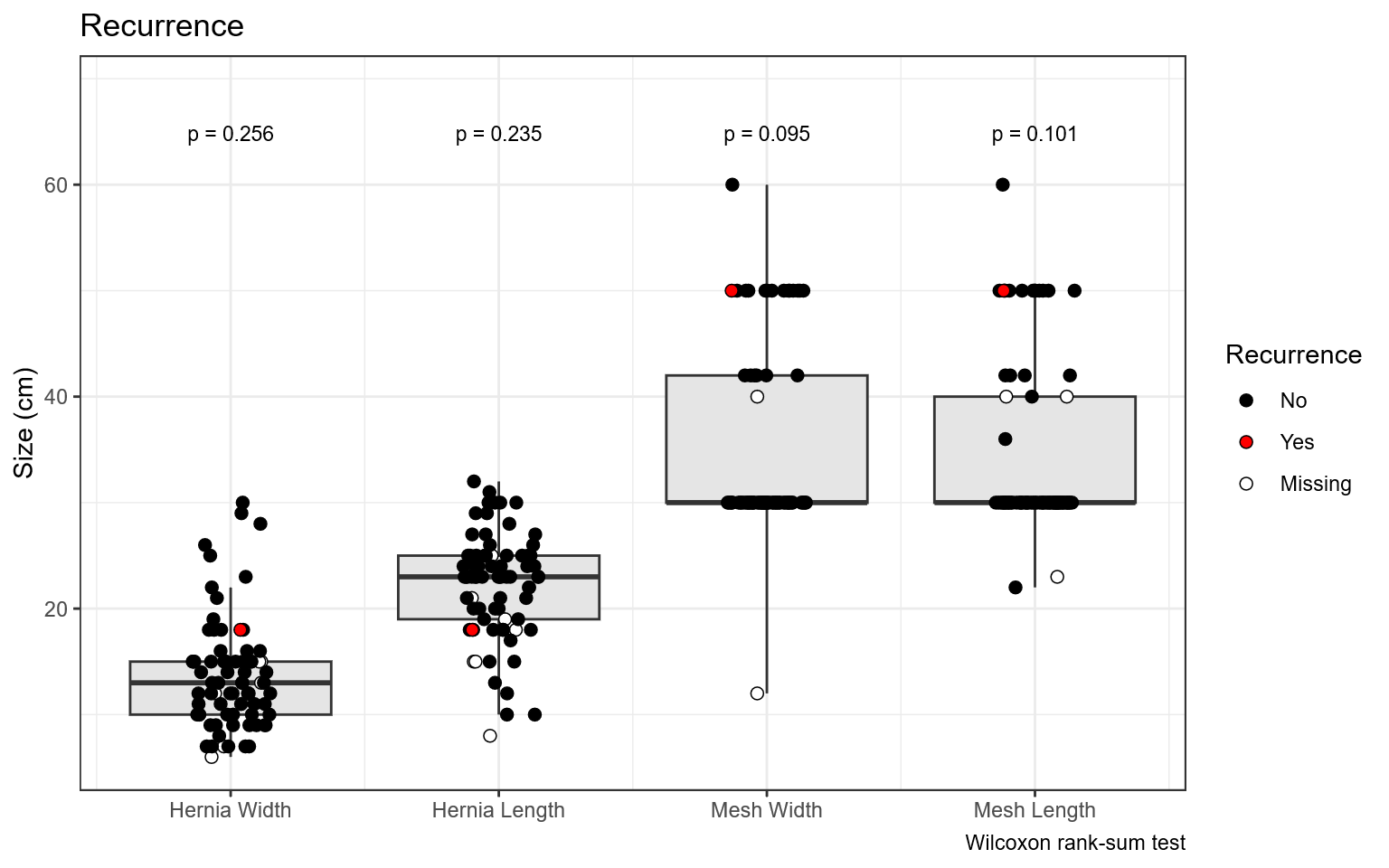


**Supplemental Figure S1. Hernia and Mesh Dimensions Stratified by Recurrence.** Distribution of hernia width, hernia length, mesh width, and mesh length (cm) across the cohort, stratified by recurrence via radiographic and clinical exam definition. Boxplots show the median and interquartile range with whiskers extending to 1.5 × IQR; individual patients are overlaid as jittered points colored red for recurrence present (n = 1), black for recurrence absent (n = 68), and white for missing data (n = 8). Point coordinates are held constant across all supplemental figures to permit direct visual comparison between outcomes. P-values shown above each measurement reflect Wilcoxon rank-sum tests comparing size distributions between patients with and without recurrence. Alpha = 0.05.


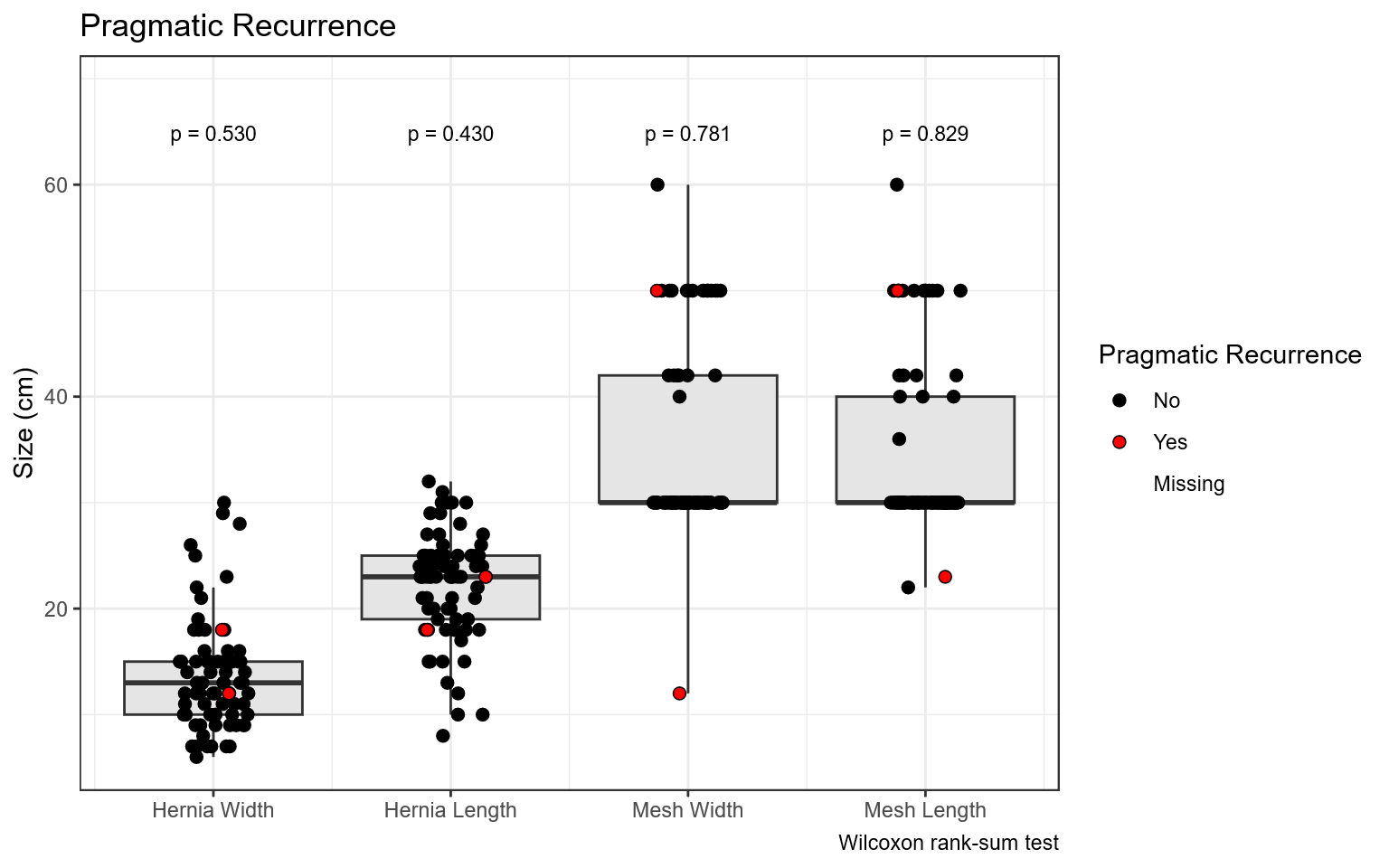


**Supplemental Figure S2. Hernia and Mesh Dimensions Stratified by Pragmatic Recurrence.** Distribution of hernia width, hernia length, mesh width, and mesh length (cm) across the cohort, stratified by pragmatic definition of recurrence. Boxplots show the median and interquartile range with whiskers extending to 1.5 × IQR; individual patients are overlaid as jittered points colored red for pragmatic recurrence present (n = 2), black for pragmatic recurrence absent (n = 75), and white for missing data (n = 0). Point coordinates are held constant across all supplemental figures to permit direct visual comparison between outcomes. P-values shown above each measurement reflect Wilcoxon rank-sum tests comparing size distributions between patients with and without pragmatic recurrence. Alpha = 0.05.


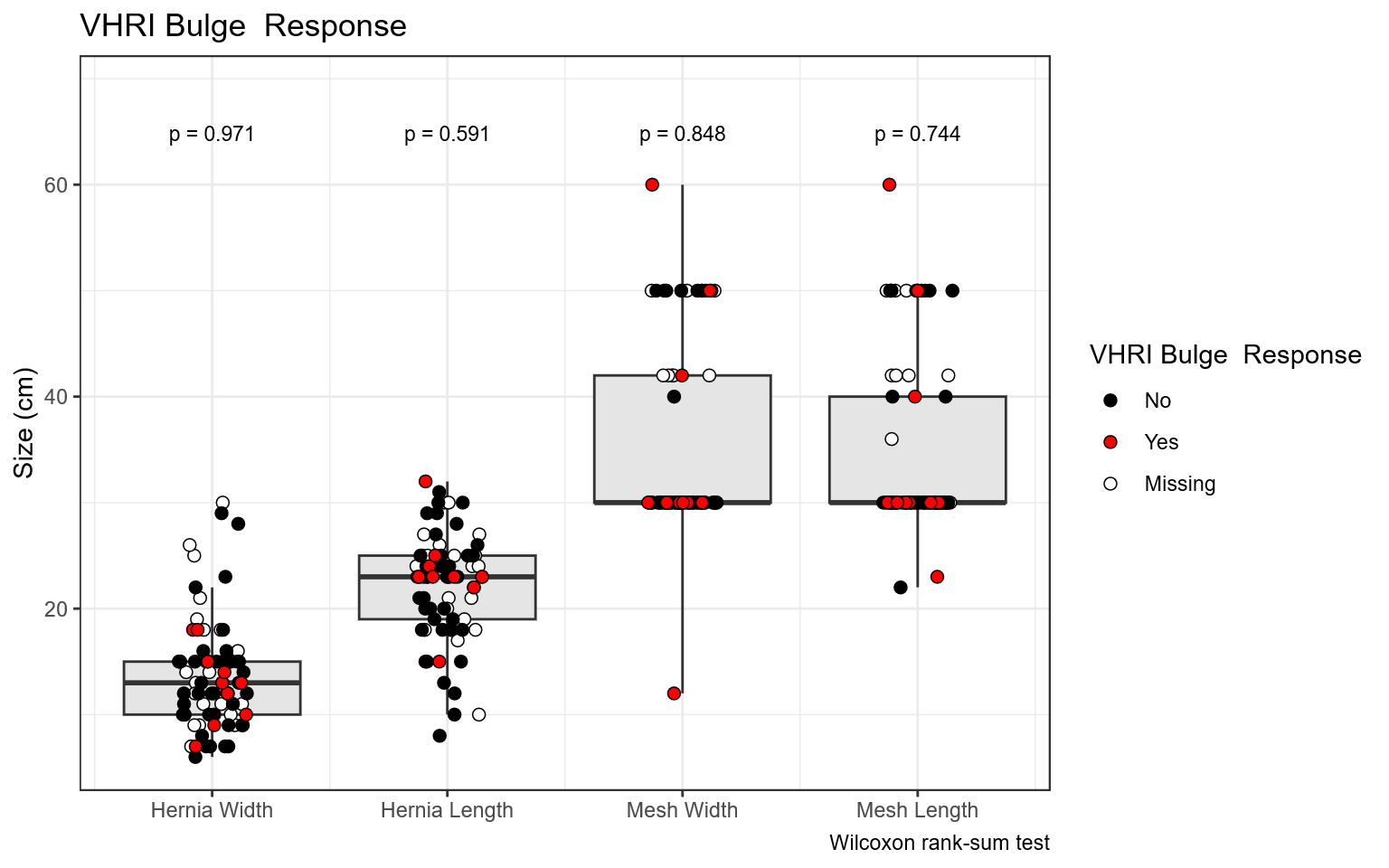


**Supplemental Figure S3. Hernia and Mesh Dimensions Stratified by Ventral hernia recurrence inventory (VHRI) Bulge-item Response.** Distribution of hernia width, hernia length, mesh width, and mesh length (cm) across the cohort, stratified by VHRI Bulge Response. Boxplots show the median and interquartile range with whiskers extending to 1.5 × IQR; individual patients are overlaid as jittered points colored red for VHRI bulge response = yes (n = 10), black for VHRI bulge response = no (n = 41), and white for missing data (n = 26). Point coordinates are held constant across all supplemental figures to permit direct visual comparison between outcomes. P-values shown above each measurement reflect Wilcoxon rank-sum tests comparing size distributions between patients with and without VHRI bulge response of yes. Alpha = 0.05.


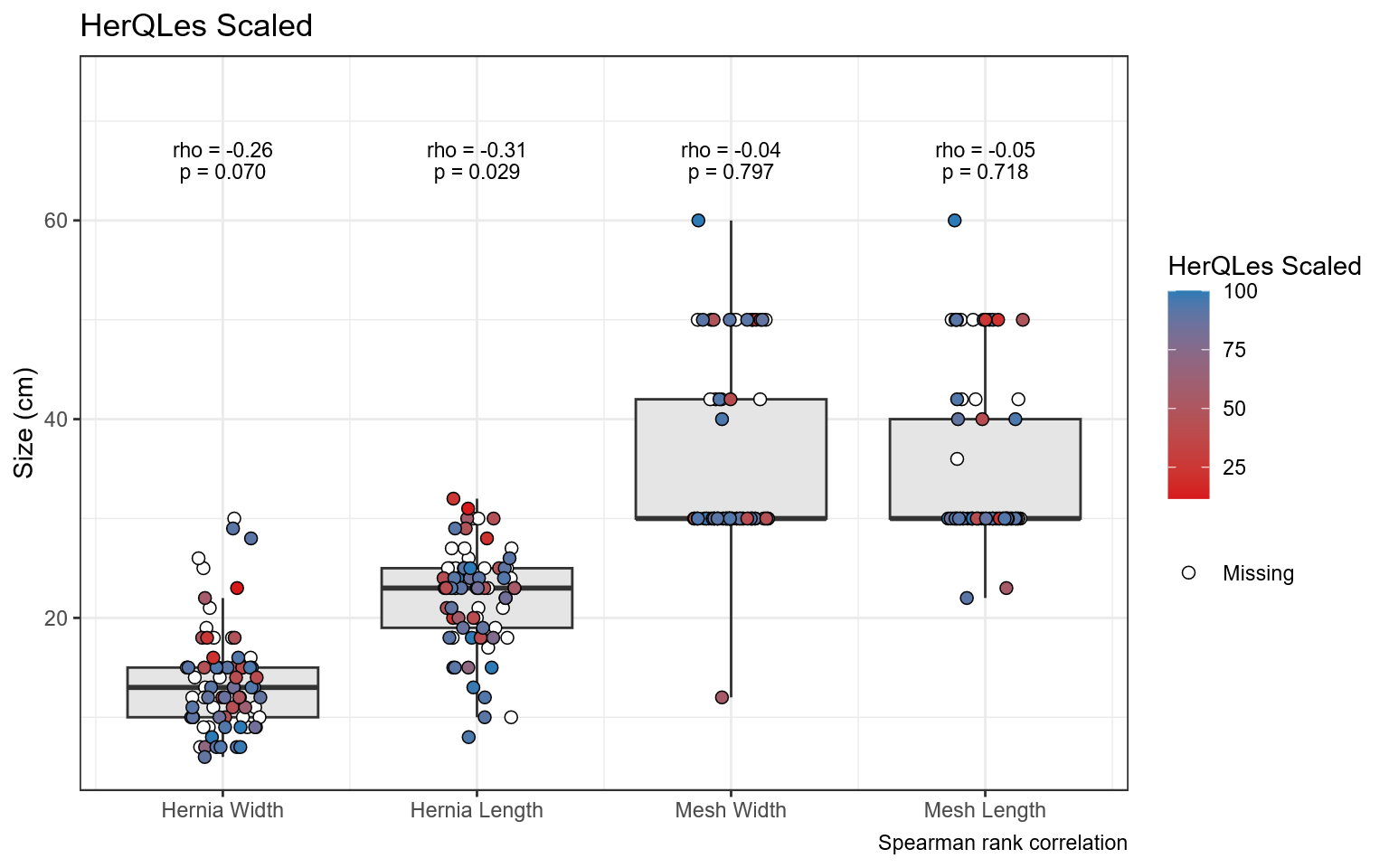


**Supplemental Figure S4. Hernia and Mesh Dimensions Stratified by HerQLes Scaled Score.** Distribution of hernia width, hernia length, mesh width, and mesh length (cm) across the cohort, with individual patients overlaid as jittered points colored by HerQLes scaled score (range 11.7–100.0; n = 49 observed, n = 28 missing). The color scale runs from red (lower scores) to blue (higher scores); missing values are shown as white points with a black outline and drawn beneath observed values. Point coordinates are held constant across all supplemental figures to permit direct visual comparison between outcomes. Spearman rank correlation coefficients (rho) and corresponding p-values shown above each measurement quantify the monotonic association between HerQLes Scaled and each size dimension. Alpha = 0.05.


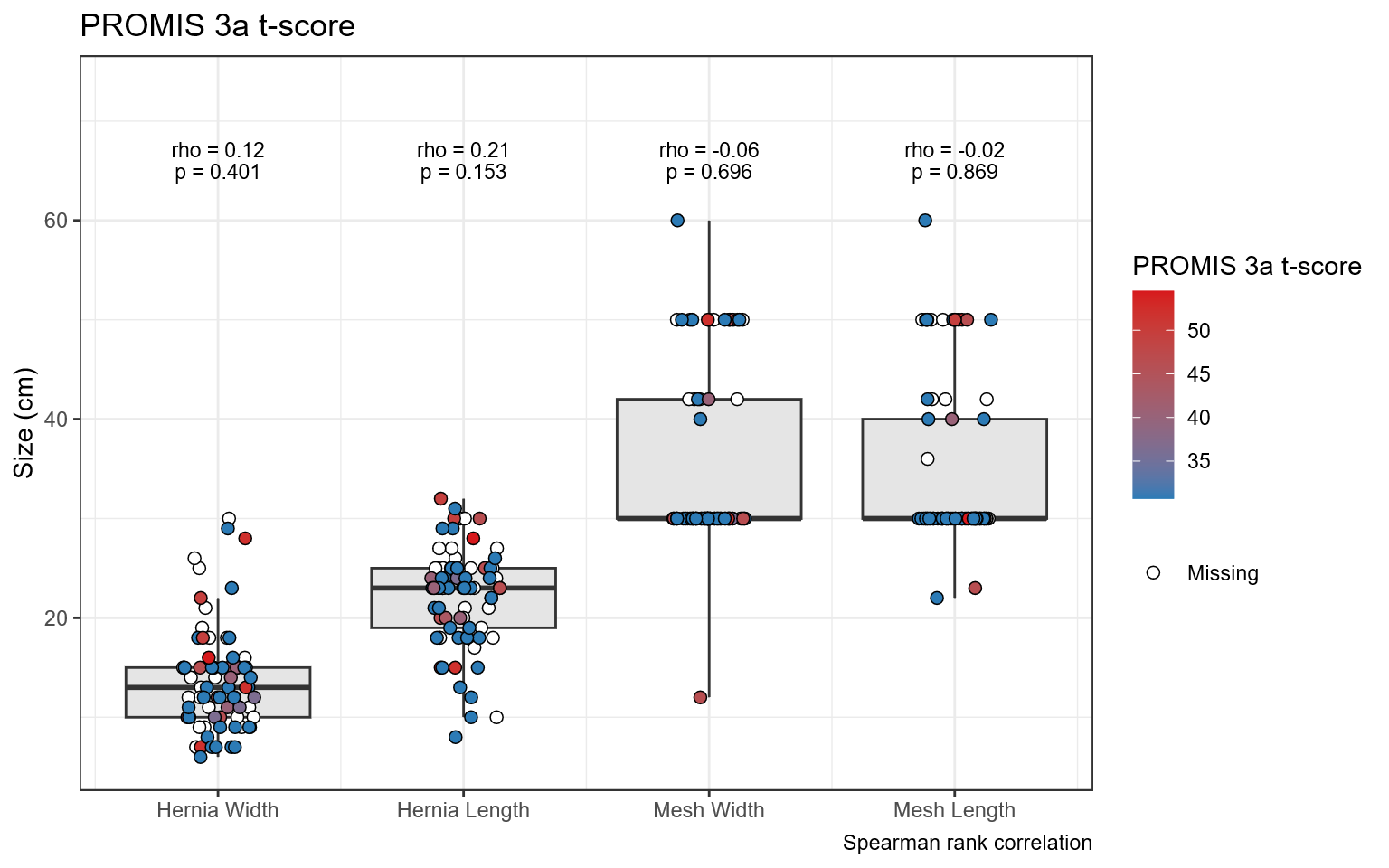


**Supplemental Figure S5. Hernia and Mesh Dimensions Stratified by PROMIS 3a t-score.** Distribution of hernia width, hernia length, mesh width, and mesh length (cm) across the cohort, with individual patients overlaid as jittered points colored by PROMIS 3a t-score (range 30.7–54.5; n = 49 observed, n = 28 missing). The color scale runs from blue (lower values) to red (higher values); missing values are shown as white points with a black outline and drawn beneath observed values. Point coordinates are held constant across all supplemental figures to permit direct visual comparison between outcomes. Spearman rank correlation coefficients (rho) and corresponding p-values shown above each measurement quantify the monotonic association between PROMIS 3a t-score and each size dimension. Alpha = 0.05.


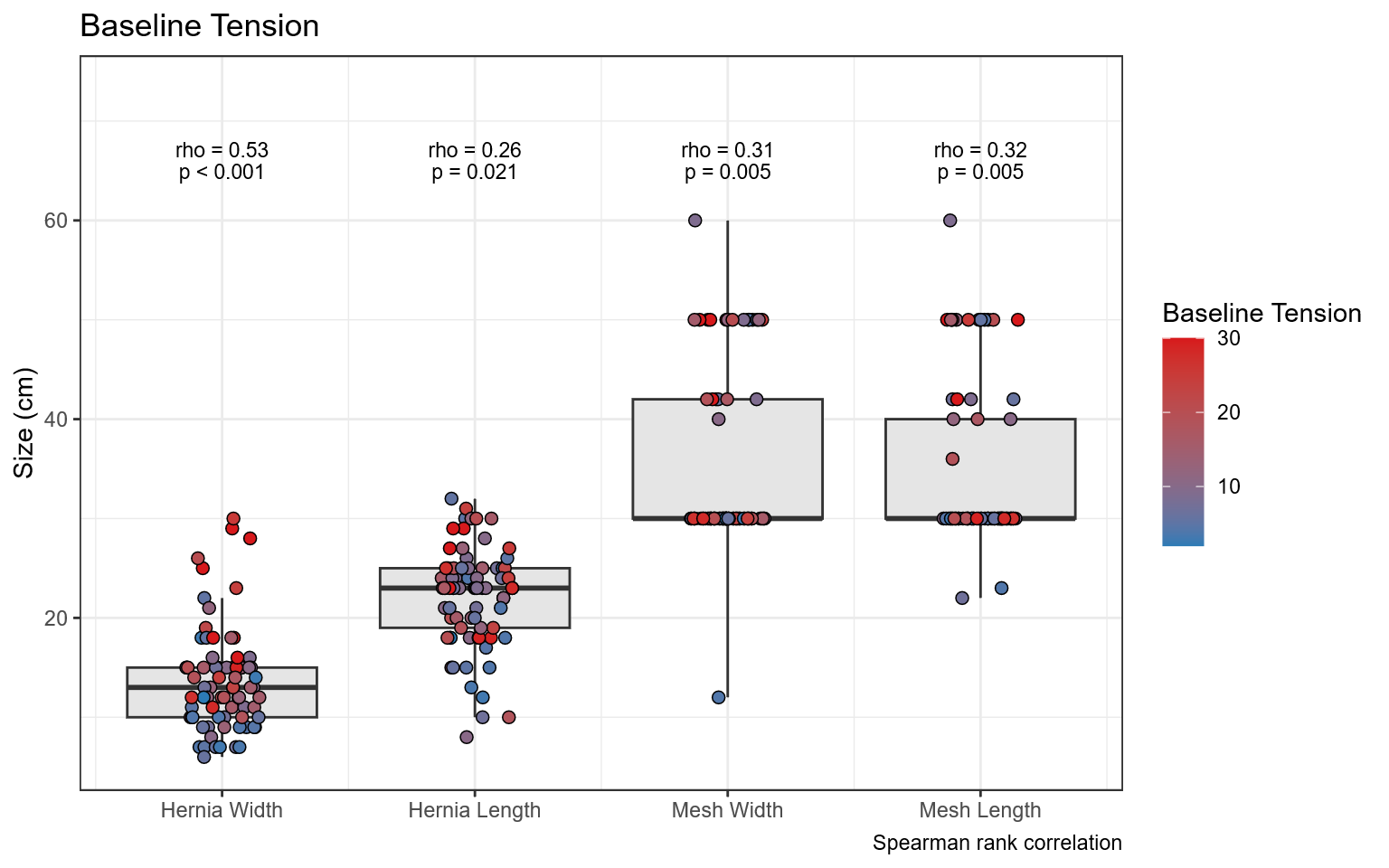


**Supplemental Figure S6. Hernia and Mesh Dimensions Stratified by Baseline Tension (lbs).** Distribution of hernia width, hernia length, mesh width, and mesh length (cm) across the cohort, with individual patients overlaid as jittered points colored by baseline anterior fascial tension (range 2.0–30.0; n = 77 observed, n = 0 missing). The color scale runs from blue (lower values) to red (higher values); missing values are shown as white points with a black outline and drawn beneath observed values. Point coordinates are held constant across all supplemental figures to permit direct visual comparison between outcomes. Spearman rank correlation coefficients (rho) and corresponding p-values shown above each measurement quantify the monotonic association between baseline tension and each size dimension. Alpha = 0.05.


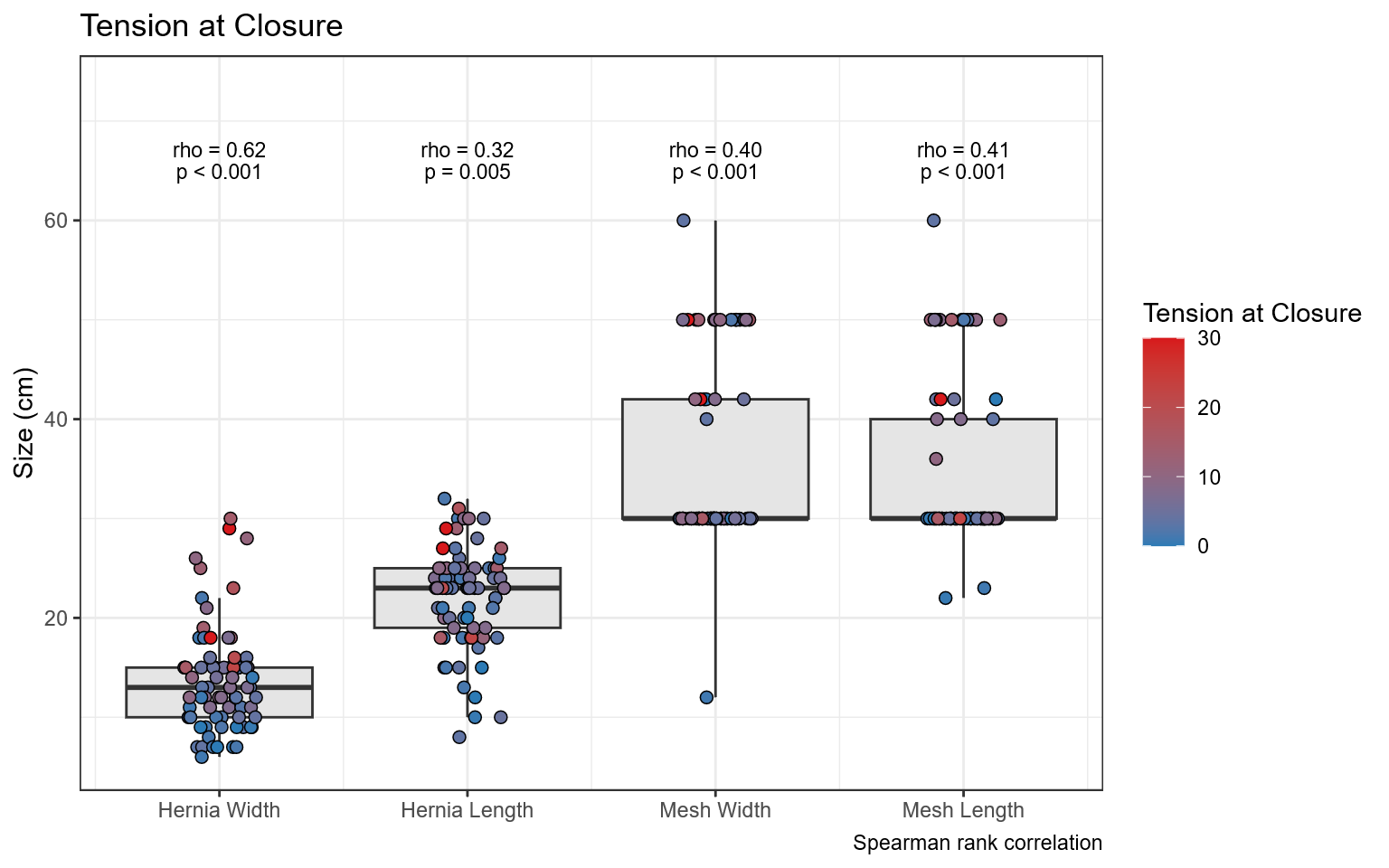


**Supplemental Figure S7. Hernia and Mesh Dimensions Stratified by Closure Tension (lbs).** Distribution of hernia width, hernia length, mesh width, and mesh length (cm) across the cohort, with individual patients overlaid as jittered points colored by anterior fascial tension after PCS-TAR immediately prior to fascial closure (range 0.0–30.0; n = 77 observed, n = 0 missing). The color scale runs from blue (lower values) to red (higher values); missing values are shown as white points with a black outline and drawn beneath observed values. Point coordinates are held constant across all supplemental figures to permit direct visual comparison between outcomes. Spearman rank correlation coefficients (rho) and corresponding p-values shown above each measurement quantify the monotonic association between closure tension and each size dimension. Alpha = 0.05.


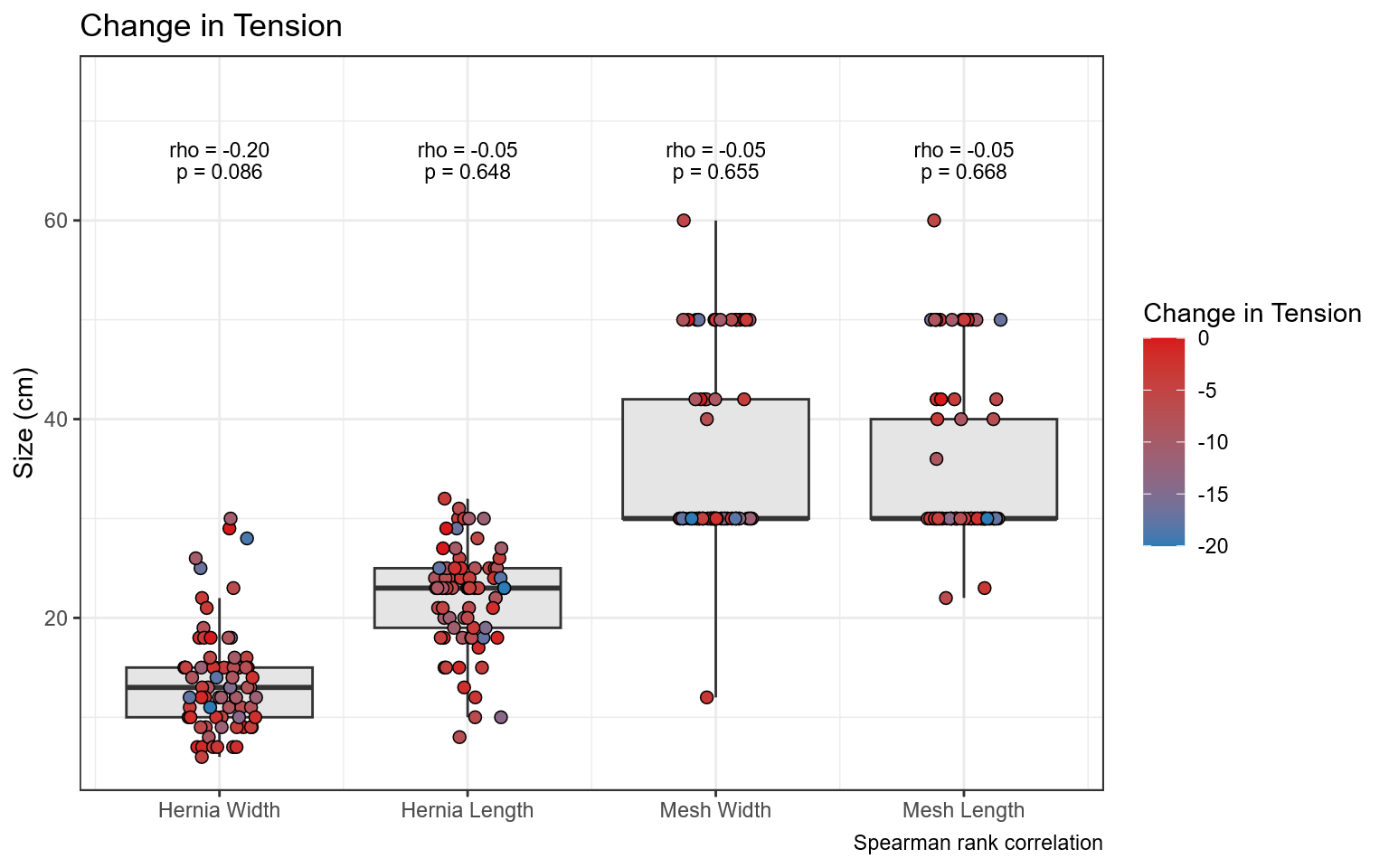


**Supplemental Figure S8. Hernia and Mesh Dimensions Stratified by Change in Tension (lbs).** Distribution of hernia width, hernia length, mesh width, and mesh length (cm) across the cohort, with individual patients overlaid as jittered points colored by change in anterior fascial tension between baseline and closure (range -20.0–0.0; n = 77 observed, n = 0 missing). The color scale runs from blue (lower values) to red (higher values); missing values are shown as white points with a black outline and drawn beneath observed values. Point coordinates are held constant across all supplemental figures to permit direct visual comparison between outcomes. Spearman rank correlation coefficients (rho) and corresponding p-values shown above each measurement quantify the monotonic association between change in tension and each size dimension. Alpha = 0.05.
